# The Causes of Cardiac Disease in Middle and Advanced Life

**Published:** 1901-03-16

**Authors:** 


### THE CAUSES OF CARDIAC DISEASE IN MIDDLE AND ADVANCED LIFE.

In the first of the Lettsomian Lectures delivered before the Medical Society Dr. Mitchell Bruce, after describing the changes to which the heart and blood-vessels are especially liable in advancing years, enumerated the influences which, after the age of 40 more particularly, threaten the heart and arteries. They may be arranged under the following heads:—Physical stress; nervous influences; extrinsic poisons, such as alcohol, tea, tobacco; disturbances of metabolism, including gout; syphilis; acute diseases, especially influenza; chronic diseases; and certain complex causes, including emphysema, which strains the right heart, Bright's disease, which strains the left, and that hereditary tendency to wear out early which causes the vital energy of the tissues of some people to become prematurely exhausted.

On the subject of physical stress Dr. Mitchell Bruce says that this is still a definite cause of cardiac and vascular damage in the second half of life, even after 70 years of age, both in the forms of sudden violent exertion and of ordinary laborious occupation. Most commonly, however, in such cases the cardiac walls are already weakened by metabolic disorders, including gout. "It is the unwise, ill-timed, ill-planned muscular exercise that injures the circulation; most often on the part of the middle-aged man who, awaking to the consciousness of growing fat and gouty, rushes inconsiderately to violent exercise for relief."

In regard to nervous influences it is to be noted that, true as it is that nervous disturbances do affect the heart and that depressing emotions do fall upon mankind more heavily as life advances, yet the connection between such nervous causes and cardio-vascular disease is often only indirect. The nervous temperament leads to overwork; worry often leads to alcohol; and in many cases worry and depression are really due to some underlying gout or influenza or the like, which are themselves the causes of any accompanying cardiac disorder.

Disturbances of metabolism including gout are by far the most prolific cause of cardio-vascular disease after 40 years of age, at any rate among the middle and higher classes in this country. This period of life brings with it comparative relaxation from work, while the pleasures of the table and ease generally are too often indulged in as a privilege of advancing years and the legitimate reward of previous years of work. The results are functional disorders of the liver, gout in regular and irregular forms, gravel, and many associated disorders. The heart and blood-vessels and kidneys are kept under constant strain, and chronic Bright's disease and cardio-vascular degeneration are not uncommonly the result.

